# Cannabidiol in the Treatment of Post-Traumatic Stress Disorder: A Case Series

**DOI:** 10.1089/acm.2018.0437

**Published:** 2019-04-09

**Authors:** Lucas Elms, Scott Shannon, Shannon Hughes, Nicole Lewis

**Affiliations:** ^1^ Rocky Vista University, Osteopathic Medical Student IV, Parker, CO.; ^2^ Department of Psychiatry, University of Colorado Denver, Denver, CO.; ^3^ School of Social Work, Colorado State University College of Health and Human Sciences, Fort Collins, CO.; ^4^ Department of Naturopathic Medicine, Wholeness Center, Fort Collins, CO.

**Keywords:** cannabidiol, cannabis, post-traumatic stress disorder, PTSD, anxiety, nightmares

## Abstract

***Objectives:*** Cannabidiol (CBD) is a non-psychotomimetic cannabinoid compound that is found in plants of the genus *Cannabis*. Preclinical research has suggested that CBD may have a beneficial effect in rodent models of post-traumatic stress disorder (PTSD). This effect is believed to be due to the action of CBD on the endocannabinoid system. CBD has seen a recent surge in research regarding its potential value in a number of neuro-psychiatric conditions. This is the first study to date examining the clinical benefit of CBD for patients with PTSD.

***Methods:*** This retrospective case series examines the effect of oral CBD administration on symptoms of PTSD in a series of 11 adult patients at an outpatient psychiatry clinic. CBD was given on an open-label, flexible dosing regimen to patients diagnosed with PTSD by a mental health professional. Patients also received routine psychiatric care, including concurrent treatment with psychiatric medications and psychotherapy. The length of the study was 8 weeks. PTSD symptom severity was assessed every 4 weeks by patient-completed PTSD Checklist for the DSM-5 (PCL-5) questionnaires.

***Results:*** From the total sample of 11 patients, 91% (*n* = 10) experienced a decrease in PTSD symptom severity, as evidenced by a lower PCL-5 score at 8 weeks than at their initial baseline. The mean total PCL-5 score decreased 28%, from a mean baseline score of 51.82 down to 37.14, after eight consecutive weeks of treatment with CBD. CBD was generally well tolerated, and no patients discontinued treatment due to side effects.

***Conclusions:*** Administration of oral CBD in addition to routine psychiatric care was associated with PTSD symptom reduction in adults with PTSD. CBD also appeared to offer relief in a subset of patients who reported frequent nightmares as a symptom of their PTSD. Additional clinical investigation, including double-blind, placebo-controlled trials, would be necessary to further substantiate the response to CBD that was observed in this study.

## Introduction

Post-traumatic stress disorder (PTSD) is a relatively common psychiatric condition with a lifetime prevalence of 6.1% in the United States.^1^ PTSD often presents in clusters of symptoms, including the re-experiencing of traumatic events through intrusive memories and nightmares, avoidance of certain distressing factors, and alterations in mood, level of arousal, and cognition. Psychotherapy is the established first-line treatment for PTSD, and various psychiatric medications are also typically employed. The development of additional treatment agents is important because current medications, including selective serotonin reuptake inhibitors, serotonin/norepinephrine reuptake inhibitors, antiadrenergic agents, and second-generation antipsychotics, have questionable efficacy and often carry significant undesirable side-effect profiles.

Although the pathophysiology of PTSD has not yet been definitively described, a number of factors are suspected to contribute to the development of this disorder. One hypothesis relates PTSD to dysregulated memory retrieval through the process of reconsolidation and impaired extinction of aversive memories.^2^ The endogenous cannabinoid system has been shown to play an important role in the process of aversive memory extinction through the activity of central CB1 receptors.^3^ Two cannabinoid receptors are known to exist in the human body: CB1 and CB2 receptors. CB1 receptors are located mainly in the brain and modulate neurotransmitter release in a manner that prevents excessive neuronal activity, thus calming and decreasing anxiety. CB1 receptors also have a role in reducing pain, inflammation, regulating movement and posture control, and regulating sensory perception, memory, and cognitive function.

Cannabidiol (CBD) is known to have multiple physiologic mechanisms of action, including 5-HT_1A_ serotonergic agonism, adenosine and opioid receptor modulation, activation of the endogenous endocannabinoid system, antagonism at GPR55 receptors, and activation of transient receptor potential channels.^4,5^ CBD's activity at 5-HT_1A_ receptors may drive its neuroprotective, antidepressive, and anxiolytic benefits, although the mechanism of action by which CBD decreases anxiety is still unclear.^6^ CBD was shown to be helpful for decreasing anxiety through a simulated public speaking test at doses of 300–600 mg in single-dose studies.^7–9^ Other studies suggest that lower doses of 10 mg/kg have a more anxiolytic effect than higher doses of 100 mg/kg in rats.^10^

Of particular interest to this study is the effect of CBD on the endogenous cannabinoid system. CBD has minimal affinity for CB1 and CB2 receptors,^11^ but it does indirectly cause activation of CB1 receptors by increasing the availability of endogenous endocannabinoids. Anandamide is an endogenous cannabinoid that acts as a partial agonist at CB1 receptors. It is metabolically deactivated by the enzyme fatty acid amide hydrolase (FAAH). CBD has been shown in some studies to inhibit FAAH, thus increasing the availability of anandamide and causing activation of the endocannabinoid system.^12^ Studies in rodent models have shown that pharmacologic activation of the endocannabinoid system through CB1-receptor agonist agents leads to decreased behavioral response to aversive memories in rodent models through the inhibition of memory reconsolidation and enhanced extinction.^13–15^ This early research suggests that agents such as CBD that cause indirect activation of the endocannabinoid system may have utility in the treatment of PTSD.

Current evidence regarding the use of CBD for PTSD in humans is minimal. One case report showed that administration of 12–37 mg of oral CBD daily was associated with a reduction in anxiety symptoms and sleep disturbances in a 10-year-old patient with PTSD due to sexual trauma.^16^ Another study showed that 32 mg of inhaled CBD resulted in consolidation of aversive memory extinction and attenuation of explicit fearful responding in healthy human subjects.^17^ See Bittencourt and Takahashi^18^ for a recent comprehensive review of pre-clinical and clinical studies regarding the relationship of CBD to PTSD. To date, no clinical trial evaluating the effectiveness of CBD in reducing symptoms of PTSD in humans has been completed.

The hypothesis of this study was that patients with DSM-5-diagnosed PTSD who were administered CBD along with routine psychiatric care would show a decrease in PTSD-specific symptomatology. This hypothesis was based on prior rodent and limited human studies that suggest that (1) CBD may cause decreased response to and increased extinction of aversive memories, and that (2) CBD may have an anxiolytic effect, which, in turn, would have therapeutic value in patients with PTSD. To this end, we conducted a retrospective file review of adult patients with PTSD who were treated with CBD as part of standard psychiatric care in an outpatient clinic. The goal of this review was to examine the tolerability of CBD and its effectiveness in PTSD symptom reduction.

## Materials and Methods

### Design and procedures

This article describes a retrospective chart review of adult psychiatric patients with a diagnosis of PTSD who consented to treatment with CBD as augmentation to routine psychiatric treatment at an outpatient psychiatric clinic. All current patients with a diagnosis of PTSD were considered for treatment with CBD between February 2016 and May 2018. Patients were not excluded based on the presence of other psychiatric comorbidities (aside from an active thought disorder) or concurrent use of cannabis. The diagnosis of PTSD was established through clinical evaluation by a mental health professional (psychiatrist, psychiatric nurse practitioner, or physician assistant). Inclusion criteria for the present analysis required a cut-off score of 33 on the Post-Traumatic Stress Disorder Checklist for the DSM-5 (PCL-5)^19^ and a minimum of two consecutive follow-up appointments after the initial intake appointment. The final sample consisted of 11 adult patients with a diagnosis of PTSD and who met inclusion criteria.

After the initial baseline assessment, PCL-5 assessments were completed by patients every 4 weeks to monitor changes in the severity of PTSD symptoms. In addition to CBD, patients also received routine treatment in the form of psychiatric medications, various psychotherapy modalities, and standard integrative treatments, as indicated for their diagnoses of PTSD and other psychiatric comorbidities. These integrative treatments often included dietary changes, herbal supplementation, neurofeedback, and intravenous infusions of vitamins and minerals.

Four patients received CBD as an oral capsule only. One patient only received CBD in the form of an oral liquid spray. Fifty-five percent (*n* = 6) of patients received both forms of CBD either concurrently or sequentially over the course of the study. The form of CBD (capsule vs. liquid spray) was determined by provider and patient preference. The CBD products used in this study were supplied by CV Sciences. Capsules were demonstrated by high-performance liquid chromatography with ultraviolet detection (HPLC-UV) to contain 22–28 mg of CBD per capsule. Patients were instructed to take whole capsules, which were assumed to contain 25 mg of CBD for dosing purposes. Patients were instructed to take liquid CBD as a specified number of sprays from a spray bottle. The liquid product used in this article was demonstrated by HPLC-UV to contain between 425 and 575 mg of CBD in total per bottle, equating to about 1.5 mg of CBD per spray.

Patients were instructed to take CBD once or twice per day based on severity of symptoms. The median starting oral capsular dose was 25 mg per day (range: 25–100). The median dose of liquid CBD given throughout the study was 9 mg per day (range: 1–16). The mean total starting dose of CBD (liquid or capsular or both) was 33.18 mg (standard deviation [SD] = 23.34). The mean total dose of CBD prescribed at the 8-week follow-up appointment at the conclusion of the study period was 48.64 mg (range: 2–100). The dose of CBD was adjusted at each 4-week appointment based on the patient's presentation and experience. Most patients received an increase in the dose of CBD because treatment was provided to maximize PTSD symptom reduction, which seemed to be directly correlated with dose. These doses are much lower than the doses used in the previous clinical literature for multiple reasons. The first is that lower doses appear to elicit an adequate clinical response in our experience. Second, the current retail cost of CBD would make the use of 600 mg per day cost-prohibitive. Finally, doses for the liquid spray route of administration are typically lower than that of capsules and are usually measured as single milligrams of CBD per spray, thus rendering higher doses impractical for patients relying on liquid CBD.

Informed consent was obtained for each patient at their intake appointment. Appointments every 4 weeks included clinical evaluation and documentation of patients' PTSD symptomatology through PCL-5 questionnaires. Concurrent psychiatric medications were held constant or changed according to routine clinical practice, whereas CBD was often intentionally used as a method of decreasing or avoiding the use of psychiatric medications. CBD was added to care, dropped from care, or refused as per individual patient and practitioner preference. The Western Institutional Review Board approved a retrospective analysis of the charts of patients with a diagnosis of PTSD who received CBD as part of their treatment program.

### Setting

Wholeness Center is a large mental health clinic with a focus on integrative medicine and psychiatry. Practitioners from a range of disciplines (psychiatry, naturopathy, acupuncture, neurofeedback, yoga, etc.) work together in a collaborative and cross-disciplinary environment. Based on existing research and patient experience, CBD had been widely incorporated into clinical care a few years before this study.

### Sample

Characteristics of the study sample are presented in Table 1. The average age of the population in this study was 39.91 (range: 22–69, *n* = 11). The majority (73%, *n* = 8) of patients were female. On average, patients were concurrently taking three psychiatric medications, including antidepressants, mood stabilizers, anxiolytics, and stimulants. One patient used cannabis daily throughout the study. Overall, 73% (*n* = 8) of patients were concurrently receiving psychotherapy as part of their overall care. Patients had on average 1.8 comorbid psychiatric conditions in addition to their PTSD diagnosis, including anxiety, mood, personality, and sleep disorders.

**Table 1.** Characteristics of the Patient Population and Concurrent Treatments Received

*Characteristic*	*Value*
Age, mean (SD)	39.91 (17.39)
Gender, *n* (%)	
Male	3 (27)
Female	8 (73)
Total psychiatric medications received, mean (SD)	3 (1.84)
Relevant medication classes received by patients, *n* (%)	
Anticonvulsant	6 (54.55)
Antidepressant	6 (54.55)
Antipsychotic	2 (18.18)
Anxiolytic/sedative	6 (54.55)
Beta-blocker	4 (36.36)
Number receiving psychotherapy, *n* (%)	8 (73)
Psychiatric comorbidities, mean (SD)	1.8 (1.54)

SD, standard deviation.

### Main outcome measures

Changes in PTSD symptoms were assessed by administering PCL-5 questionnaires before starting CBD and at 4-week intervals thereafter. The PCL-5 is a reliable and valid test published in 2013 to provide an assessment of PTSD symptoms that is consistent with changes to the criteria of PTSD in the DSM-5.^20^ Patients complete a 20-question self-report form that rates their symptoms on a scale of 0–4, with total scores ranging from 0 to 80. The PCL-5 is used only to assess PTSD symptom severity and no particular numeric score establishes a definitive diagnosis of PTSD, although a provisional diagnosis of PTSD may be established based on (1) individual scoring clusters in relation to the diagnostic criteria of the DSM-5 or (2) a cumulative score of 33 or greater. Side effects and tolerability were assessed through spontaneous patient self-report and were documented in case records accordingly. Any other spontaneous comments or complaints of patients were also documented in case records and included in this analysis.

### Analysis

After patient information was de-identified, the main outcome measure of total PCL-5 score was tracked and descriptively analyzed based on mean score and percentage change from the prior 4-week appointment. The endpoint for statistical analysis of the study was set at 8 weeks after initial evaluation. Data beyond this period were not analyzed because follow-up beyond this period dropped to <50%. However, anecdotal data were still considered and qualitatively presented in the Results sub-section.

## Results

Of the initial 21 patients who were prescribed CBD and attended at least one follow-up appointment, 86% (*n* = 18) remained on CBD and completed PCL-5 questionnaires at 4 weeks of follow-up. This number dropped to 67% (*n* = 14) at 8 weeks of follow-up. This is generally reflective of normal attrition rates experienced in this clinic, and reasons for discontinuation of care were largely unknown. Of the 14 patients who remained in the study at the endpoint of 8 weeks, 11 met inclusion criteria for data analysis by attending both 4- and 8-week follow-up appointments. Attrition rates by week are depicted in Table 2.

**Table 2.** Number of Patients Who Presented at the Corresponding Four-Week Interval Appointment After Their Intake and Completed a PCL-5 Questionnaire

*Weeks in study*	*Number of patients (% of initial sample)*
Intake	21 (100)
4	18 (86)
8	14 (67)
12	8 (38)
16	6 (29)
20	7 (33)
24	5 (24)
28	4 (19)
32	4 (19)
36	4 (19)
40	3 (14)

PCL-5, Post-Traumatic Stress Disorder Checklist for the DSM-5.

**FIG. 1.**
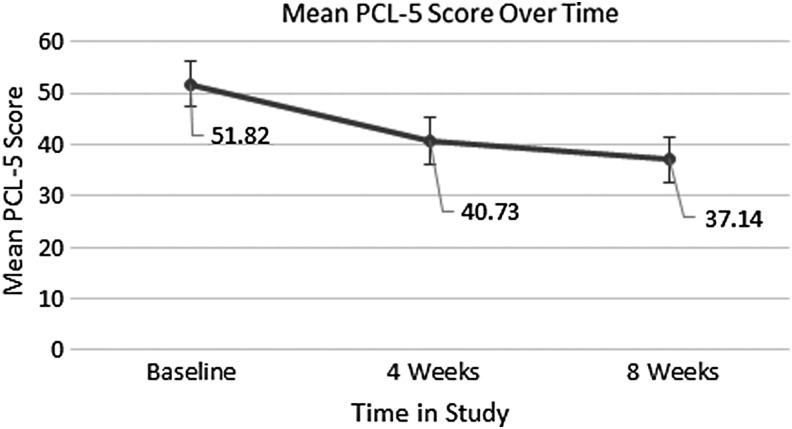
Mean PCL-5 score of the sample over the course of the study depicted as a function of time showing the observed decrease. PCL-5, Post-Traumatic Stress Disorder Checklist for the DSM-5.

The baseline mean PCL-5 score for the statistical analysis sample was 51.82 (SD = 9.13). After 4 weeks of treatment, 91% (*n* = 10) of patients from this group reported a decrease in symptoms of PTSD. PCL-5 scores declined from 51.82 to 40.73 (SD = 12.92), a decrease of 21%. One patient did, however, experience an increase in PCL-5 score from 51 to 63. After 8 weeks of treatment with CBD, 73% (*n* = 8) of patients reported a further decrease in PTSD symptoms from their follow-up appointment 4 weeks earlier, and the average PCL-5 score decreased 9% down to 37.14 (SD = 14.38). At 8 weeks, 27% (*n* = 3) of patients had worsening of PTSD symptoms from their prior 4-week appointment, with a mean score increase of 8 (SD = 6.08). Despite this increase from the prior appointment experienced by some patients, nearly all patients (*n* = 10) reported a PCL-5 score that was lower than their baseline at the beginning of the study, with an average decrease of 28%. The results of the study are depicted graphically in Figure 1.

Four patients from the initial sample continued to receive CBD for 36 weeks or more. These patients had an initial mean PCL-5 score of 57.75 (SD = 6.20) and all experienced long-term sustained decreases in PCL-5 scores, with a mean score at 36 weeks of 29.25 (SD = 9.88). An unexpected finding revealed through the course of this analysis was a possible effect of CBD on nightmares. Item number 2 on the PCL-5 questionnaire assesses symptoms of “repeated, disturbing dreams of the stressful experience.” Of the patients who responded to item number 2 on the PCL-5 with a severity rating of 3 (“quite a bit”) or higher, 50% (*n* = 4) reported a subjective improvement in their nightmares after starting CBD. Of the total 21 patients in the original sample, 38% (*n* = 8) also reported subjective improvement in the quality of their sleep. Other reported benefits of CBD included decreased anxiety (*n* = 3), improved focus (*n* = 1), and improved mood (*n* = 1).

CBD was well tolerated by the majority (76%, *n* = 16) of the total number of patients (*n* = 21). Two patients reported feeling fatigue after taking CBD. One patient experienced daytime fogginess and impaired concentration the day after a night-time CBD dose. Two patients reported gastrointestinal bloating or pain. One of these patients had pre-existing inflammatory bowel syndrome and anorexia. One patient with a history of gastroesophageal reflux reported worsening reflux symptoms.

## Discussion

Patients taking daily oral CBD over an 8-week period demonstrated an overall decrease in PTSD symptom severity as measured by continual decreases in mean PCL-5 scores. CBD was well tolerated and no patients discontinued it due to side effects, although a minority of patients did report fatigue and gastrointestinal discomfort. It is unclear whether the fatigue caused by CBD is due to a sedative effect, and further information should be obtained about the safety profile of CBD.

Doses of CBD used in this study were generally lower than those used in prior preclinical and clinical research. Patients did generally report greater improvement in symptoms with higher doses of CBD. Further investigation into the optimal dosing of CBD for PTSD is warranted. Patients also received liquid spray and oral capsular forms of CBD, and it is unknown whether there is a difference in response between the two routes of administration. CBD is commercially available in many different forms, and further studies should be done to determine the most effective form of CBD.

A surprising number of patients with significant symptomatology related to PTSD nightmares reported subjective improvement in these symptoms. It is unclear whether this is due solely to the placebo effect or an effect of CBD based on a previously unknown mechanism of action. Due to the current paucity of medications approved for PTSD nightmares, further investigations should examine whether CBD has a clinical benefit for patients with PTSD-related nightmares.

Although a qualitative examination of the results of this study appears to show a sustained decrease in PCL-5 score after extended periods, the early endpoint for statistical analysis makes it difficult to definitively determine whether continued use of CBD results in continued improvement of symptoms. We have shown that the mean PCL-5 score decreased at every 4-week interval for the majority of patients at the 4- and 8-week follow-ups, but further work should be done to determine how long this effect continues and whether there is an eventual reversal and return to baseline.

### Limitations

The results of this study should be interpreted carefully as this was a retrospective, open-label chart review and, as such, does not include a placebo or a control group. Concurrent psychiatric medications were frequently added, removed, and changed through the course of the study. A precise dosing system for CBD was not established, and regimens varied between patients. Patients also received liquid or oral capsular CBD without a definitive guideline, and typical doses between the two routes of administration differed widely. Although the product was derived from agricultural hemp, it may still contain trace amounts of delta-9-tetrahydrocannabinol. The sample used in this study was small in size, consisting of only 11 patients, and was disproportionately female. Significant attrition was observed and some patients could not be included based on non-consecutive appointments, further reducing the sample size and limiting the endpoint of the analysis to 8 weeks after initial evaluation. The study was conducted at a clinic with a focus on integrative medicine, and the patient population may differ at baseline from the general psychiatric population. There may also be a selection bias among this patient population as patients at this clinic often seek to avoid the use of psychiatric medications. The role of cannabis in society and patients' pre-existing beliefs may have played a role in the way that patients experienced CBD and could plausibly contribute to an increased placebo effect. Randomized, controlled studies are clearly indicated to fully explore the benefit of CBD for symptoms of PTSD that was observed in this chart review.

## Conclusions

This retrospective chart review represents the first case series, to our knowledge, examining the effect of CBD on symptoms of PTSD in humans. The results indicate that oral CBD, when given in addition to routine outpatient psychiatric care, may have a beneficial effect for patients with PTSD.

## About Cannabidiol

CBD is a phytocannabinoid compound found in plants of the genus *Cannabis*. It is not responsible for the psychotomimetic effect traditionally associated with cannabis use, as this is attributed to another phytocannabinoid: delta-9-tetrahydrocannabinol (delta-9-THC). Different varieties of cannabis may have differing percentages of CBD and delta-9-THC. Although CBD can be derived from cannabis plants that are grown for recreational use, the CBD used in this study was derived from agricultural hemp, a cannabis plant that contains <0.3% delta-9-THC. The process of extracting CBD oil yields not only a product containing almost entirely CBD but also other cannabinoids in trace amounts, and it is not completely free of delta-9-THC. CBD remains in a legal gray area and there have recently been frequent changes to policy. Although law varies from state to state, CBD remains federally illegal and categorized by the Drug Enforcement Administration as a Schedule I substance.
